# Quantitative proteomic analysis of cerebrospinal fluid reveals CD163, A2M and full-length APP as potential diagnostic biomarkers of paediatric bacterial meningitis

**DOI:** 10.1186/s12953-022-00191-5

**Published:** 2022-05-06

**Authors:** Ting Luo, Sai Yang, Yan Chen, Shulei Liu, Liming Yang, Nanfei Hu, Ye Ma, Jun Qiu, Kewei Wang, Liping Li, Lihong Tan

**Affiliations:** 1 Pediatrics Research Institute of Hunan Province, Hunan Children’s Hospital, Changsha, China; 2 Department of Neurology, Hunan Children’s Hospital, Changsha, China

**Keywords:** Paediatric bacterial meningitis, Cerebrospinal fluid, Diagnostic biomarkers, Proteomics, Infection of the central nervous system

## Abstract

**Background:**

Bacterial meningitis (BM) is a life-threatening infectious disease of the central nervous system in infants and children. To date, no diagnostic methods for the early and precise diagnosis of paediatric BM have been developed.

**Methods:**

A label-free cerebrospinal fluid (CSF) quantitative proteomic analysis of 8 patients with confirmed or suspected BM, 9 patients with confirmed or suspected viral meningitis (VM) and 6 non-CNS-infected hospital patients was performed via high-resolution LC–MS/MS.

**Results:**

Our CSF proteomic analysis allowed the identification of critical differences between the BM and non-BM groups. Compared to the proteomes of the non-BM groups, the proteome of the paediatric BM group was characterized by upregulation of complement and coagulation cascades, regulation of IGF transport, uptake by IGF-binding proteins and acute inflammatory response, downregulation of developmental growth, and metabolism of carbohydrates. Moreover, the levels of CD163, A2M and full-length APP in CSF showed excellent diagnostic performance for paediatric BM, with AUC values of 0.911 (95% CI: 0.839–0.984), 0.908 (95% CI: 0.816–1.000) and 0.944 (95% CI: 0.86, 1.000), respectively. Among them, A2M and full-length APP are reported here for the first time as potential diagnostic biomarkers of BM. The findings imply that peptidase regulator activity plays an important role in BM and provide potential novel targets for precision medicine in paediatric BM.

**Conclusions:**

CD163, A2M and full-length APP are validated as potential diagnostic biomarkers of paediatric BM.

**Supplementary Information:**

The online version contains supplementary material available at 10.1186/s12953-022-00191-5.

## Background

Meningitis is a life-threatening infectious disease of the central nervous system and is the third leading cause of death in newborns and a major cause of death in infants [1]. The mortality of meningitis approaches 10% in infants, especially preterm and chronically hospitalized infants. Moreover, approximately 20–50% of survivors develop seizures, cognitive deficiencies, motor abnormalities, and hearing and visual impairments [2, 3]. Different aetiological agents lead to varied severities and outcomes of meningitis [4]. In comparison to viral meningitis (VM), bacterial meningitis (BM) is more commonly acute, and it can rapidly develop into fatal disease. Moreover, more than 15% of survivors have been found to have permanent neurological sequelae, such as hearing impairment, developmental delay and cognitive deficiencies, and a high rate of disability [5]. Therefore, the early diagnosis of BM in infants is especially crucial for effective treatment.

For the diagnosis of BM, culture of cerebrospinal fluid (CSF) is the traditional gold standard. However, this process is time-consuming and has low sensitivity. After empirical antibiotic treatment, CSF culture and blood culture can easily produce false-negative results. In several studies, up to one-third of infants with BM had negative blood cultures [6–8]. In addition, routine diagnosis of CSF parameters alone, such as white blood cell count, glucose level, and protein level, is not sufficiently sensitive and specific for the diagnosis of BM.

A series of diagnostic biomarkers for BM have been identified, including C-reactive protein (CRP), procalcitonin (PCT) and vitamin D-binding protein. However, none of these biomarkers alone provide sufficient sensitivity or specificity for the diagnosis of BM [9–11]. In recent years, mass spectrometry (MS) has been widely used to screen biomarkers of meningitis. CSF kininogen-1 has been identified as a potential biomarker to distinguish streptococcal pneumonia from meningococcal meningitis via 2D-PAGE analysis [12]. Using a label-free LC–MS/MS method, Carrol et al*.* identified CSF S100 A9, myeloperoxidase, cathelicidin, ceruloplasmin and cystatin C as potential diagnostic and therapeutic targets of paediatric pneumococcal meningitis [13]. In addition, GFAP and sAPPα/β analysed via 2D-DIGE have been reported to be highly specific diagnostic markers of adult BM [14]. However, 2D electrophoresis-based proteomic methods are less sensitive for protein detection than MS methods.. In addition, the many suspected meningitis patients who have clinical symptoms but lack microbial diagnosis confirmation due to a low positive rate of CSF culture are not represented in these studies. Therefore, the identified diagnostic biomarkers are usually not suitable for suspected meningitis and cannot provide guidance for precise and early treatment under uncertain conditions.

In this study, a label-free CSF quantitative proteomic analysis of 8 patients with confirmed or suspected BM, 9 patients with confirmed or suspected VM and 6 non-CNS-infected hospital patients was performed by high-resolution LC–MS/MS. By comparing protein expression in patients with BM with that in patients with VM and hospital controls, we aimed to identify characteristic biological processes in BM and potential diagnostic biomarkers for paediatric BM, which may contribute to early diagnosis and precise treatment.

## Methods

### Patient recruitment and sample collection

**Fig. 1:**
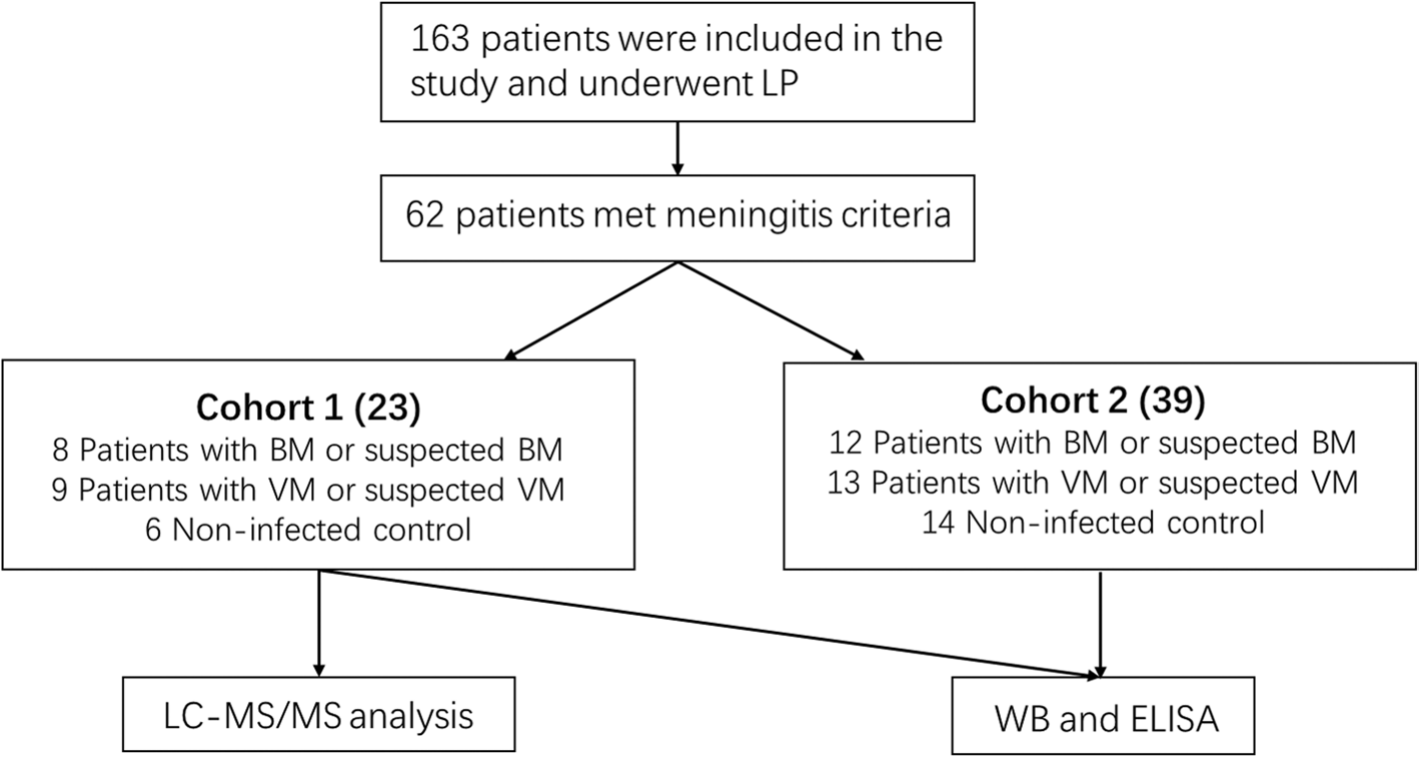
Workflow of the study design

Between June 2019 and August 2020, 163 paediatric patients admitted to the Department of Pediatric Neurology of Hunan Children’s Hospital were recruited for enrolment into the paediatric meningitis cohort of Hunan Children’s Hospital. The criteria for patient inclusion into the BM, VM and control groups were based in part on the European Society for Clinical Microbiology and Infectious Diseases (ESCMID) guidelines for the diagnosis and treatment of acute BM, and the details are presented in Table 1 [15]. Samples from a total of 62 patients were selected for inclusion in this study. Cohort 1 consisted of 8 patients with BM (representing 3 confirmed cases and 5 suspected cases), 9 patients with VM (representing 2 confirmed cases and 7 suspected cases) and 6 noninfected patients and was selected for CSF proteome analysis. Cohort 2 was composed of the remaining 12 patients with confirmed or suspected BM, 13 patients with confirmed or suspected VM and 14 hospital noninfected controls. Samples from these patients were used in enzyme-linked immunosorbent assay (ELISA) tests and western blots for validation. The workflow of patient screening is shown in Fig. 1. All involved patients underwent lumbar puncture for CSF. For research purposes, ca. 500 μl of CSF sample per patient was prepared within 2 h after collection and stored at -80 °C. Data on demographic and clinical characteristics, diagnosis and laboratory test results were extracted from electronic medical records. The study protocol was reviewed and approved by the Medical Ethics Committee of Hunan Children’s Hospital. Informed consent for sample collection was obtained from the parents or legal guardians of the patients.

**Table 1:** The inclusion criteria for the paediatric meningitis cohort in this study

Inclusion Criteria
**1. Bacterial meningitis group**
a) Bacterial meningitis: Samples with positive gram staining, culture or PCR
b) Suspected bacterial meningitis: at least 4 of the following are present: fever, rash, neurologic impairment/seizures or altered mental status, CSF protein > 0.5 g/L, CSF glucose < 2.8 mmol/L, and CSF WBC > 20
**2. Viral meningitis group**
a) Viral meningitis: Samples with positive PCR or anti-virus antibody test
b) Suspected viral meningitis: Presence of clinical symptoms of meningitis without bacterial characteristics (as mentioned in 1b)
**3. Noninfected control group**
Absence of CNS infection. Here, benign intracranial hypertension, febrile convulsion and acute cerebellar ataxia were mainly involved

### Protein digestion

Protein digestion was performed using the in-solution digestion method. Briefly, 50 μl of CSF sample was added to 6 μl of 1% Rapigest SF surfactant (Waters, USA) in 50 mM NH_4_HCO_3_ and incubated at 60 °C for 30 min. Then, DTT at a final concentration of 10 mM was added to each sample, which was incubated at 56 °C for 45 min of reduction reactions and then subjected to alkylation with IAM at a final concentration of 50 mM for 30 min at room temperature in the dark. Next, digestion was conducted with trypsin (Promega, USA) at 37 °C overnight, and the amount of trypsin was provided at a ratio of 30 to 1 (protein:trypsin). Formic acid was added to a final concentration of 0.5% to each sample, which was incubated at 37 °C for 50 min to terminate protein digestion and then centrifuged at 13,000 rpm for 10 min. The resulting peptides were desalted, and their concentrations were determined by BCA peptide assay. The remaining peptide samples were subjected to lyophilization.

### LC–MS/MS analysis

LC–MS/MS analysis was performed on an EASY-nLC 1000 UHPLC system (Thermo Scientific, USA) coupled to a Q ExactiveTM Plus mass spectrometer. First, 500 ng peptide digests were resolved in mobile phase A (0.1% formic acid in 2% ACN-water) and directly loaded onto a homemade reversed-phase analytical column (15 cm length, 75 μm i.d.). The peptides were eluted at a flow rate of 500 nl/min in mobile phase B (0.1% formic acid in 90% ACN-water) with the following gradient: 0–90 min, 5% ~ 25% B; 90–112 min, 25% ~ 35% B; 112–116 min, 35% ~ 80% B; and 116–120 min, 80% B. Next, the peptides were injected into an NSI ion source for ionization with an ion spray voltage of 2.1 kV and analysed on a Q Exactive™ Plus mass spectrometer. Data-dependent acquisition (DDA) mass spectrum techniques were used to acquire tandem MS data. For DDA, the mass spectrometer was operated with an Orbitrap at a resolution of 70,000 and a mass range of 350–1800 m/z. Then, as identified according to signal intensity, the top ten precursor ions of peptides were selected for MS/MS using high-energy collision dissociation (HCD) and operated with an Orbitrap at a resolution of 17,500. To improve the efficiency of MS, the automatic gain control (AGC) was set to 5E4, the signal threshold was set to 40,000 ions/s, the maximum injection time was set to 100 ms, and the dynamic exclusion time of tandem MS was set to 30 s to avoid repeated scanning of precursor ions.

### Database search

The resulting MS/MS data were processed using the MaxQuant search engine (v.1.5.2.8). Tandem mass spectra were searched against the SwissProt (human) database concatenated with the reverse decoy database. Trypsin/P was specified as a cleavage enzyme, with up to 2 missing cleavages allowed. The mass tolerance for precursor ions was set as 20 ppm in the first search and 5 ppm in the main search, and the mass tolerance for fragment ions was set as 0.02 Da. Carbamidomethyl on Cys was specified as a fixed modification, and oxidation on Met was specified as a variable modification. The FDR was adjusted to < 1%, and the minimum score for peptides was set as > 40.

### Bioinformatics analysis

#### Principal component analysis (PCA)

Based on the protein expression levels of all 23 CSF samples, PCA was performed by applying Origin software with the PCA plugin. A two-dimensional PCA scatter plot was constructed to visualize the distribution of all samples using quantified proteins. A shorter distance between two samples indicated higher similarity.

#### Identification of differentially expressed proteins (DEPs) between the BM and non-BM groups

Protein expression levels were quantified based on the MS signal intensity of each protein in each sample using a label-free method. First, the standardized relative intensity of the protein was multiplied by the initial protein concentration of the CSF sample to obtain the relative intensity per CSF volume of the protein. The average relative intensity value of the group was calculated, and then the ratio of the average value between two groups was calculated, which was used as the final differential expression of the comparison group. Second, the relative quantitative value of the protein in each patient was log2-transformed (so that the data conformed to a normal distribution), and then the P value was calculated by the two-sample two-tailed t test method. Proteins were considered significantly differentially regulated at *p* value < 0.05 and a fold change threshold of more than 1.5 or less than 1/1.5. Furthermore, a protein needed to be expressed in at least half of the samples within each comparison group.

#### Heatmap of DEPs in patient samples

A heatmap of the expression levels of 171 significantly DEPs with hierarchical clustering was generated using the “HeatMap” tool of Tbtools v1.09852 software. Tbtools is a toolbox for biologists that integrates various biological data-handling tools with a user-friendly interface [16].

#### Gene Ontology and pathway enrichment analyses

The enrichment of functions and pathways of all the significantly DEPs between the BM and non-BM groups was analysed via the Metascape online website, which is an integrated portal consisting of functional enrichment, interactome analysis, gene annotation, and membership search to leverage over 40 independent knowledge bases. Terms with a corrected *p* value < 0.05 were considered significant.

#### Visualization of the network of enriched functions

The network of enriched functions and pathways was visualized by Cytoscape (version 3.8.2) with the plugins ClueGO and CluePedia.

##### ELISA

The concentrations of CD163, alpha 2-macroglobulin (A2M) and sAPPα in CSF in the samples of cohort 1 and cohort 2 in this study were analysed using the Human CD163 Quantikine ELISA Kit (DC1630, R&D Systems), the human alpha 2-macroglobulin ELISA kit (ab108888) and the Human APP ELISA Kit (KHB0051, Invitrogen), respectively. Measurements were performed in accordance with the manufacturers’ instructions.

## Results

### Study design

In this study, a label-free CSF quantitative proteomic analysis of 6 samples from noninfected hospital controls, 8 samples from patients with confirmed or suspected BM and 9 samples from patients with confirmed or suspected VM was performed by high-resolution LC–MS/MS. The clinical characteristics of the patients in this study are detailed in Table 2.

**Table 2:** Baseline clinical characteristics of the included patients in this study

	**BM group**	**VM group**	**Noninfected control group**
**Gender**			
Female	6	7	10
Male	14	15	10
**Age (months)**			
Median (range)	0.25 (0.083–12.75)	6.5 (0.5–12.83)	1.125 (0.42–5.33)
**CSF parameters**			
Median (range)			
WBC	73 (1–344)	30 (1–360)	4 (0–18)
%PMN	0.2 (0.05–0.8)	0.1 (0.05–0.4)	
Glucose (mmol/L)	2.035 (0.25–4.03)	2.845 (2.29–4.72)	2.91 (2.56–4.3)
Protein (g/L)	0.535 (0.08–1.62)	0.165 (0.02–0.53)	0.15 (0.03–0.29)
**Bacterial culture**			
Blood	6	/	/
CSF	4	/	/
**Virus**			
PCR	1	2	/
Virus antibody test	7	16	5
**Clinical characteristics**			
Fever	19	20	14
Cough	5	4	10
Vomiting	8	13	3
Diarrhoea	4	1	2
Headache	5	13	2
Meningeal irritation	3	3	0

### The CSF protein profiles of the BM, VM and control groups

**Fig. 2:**
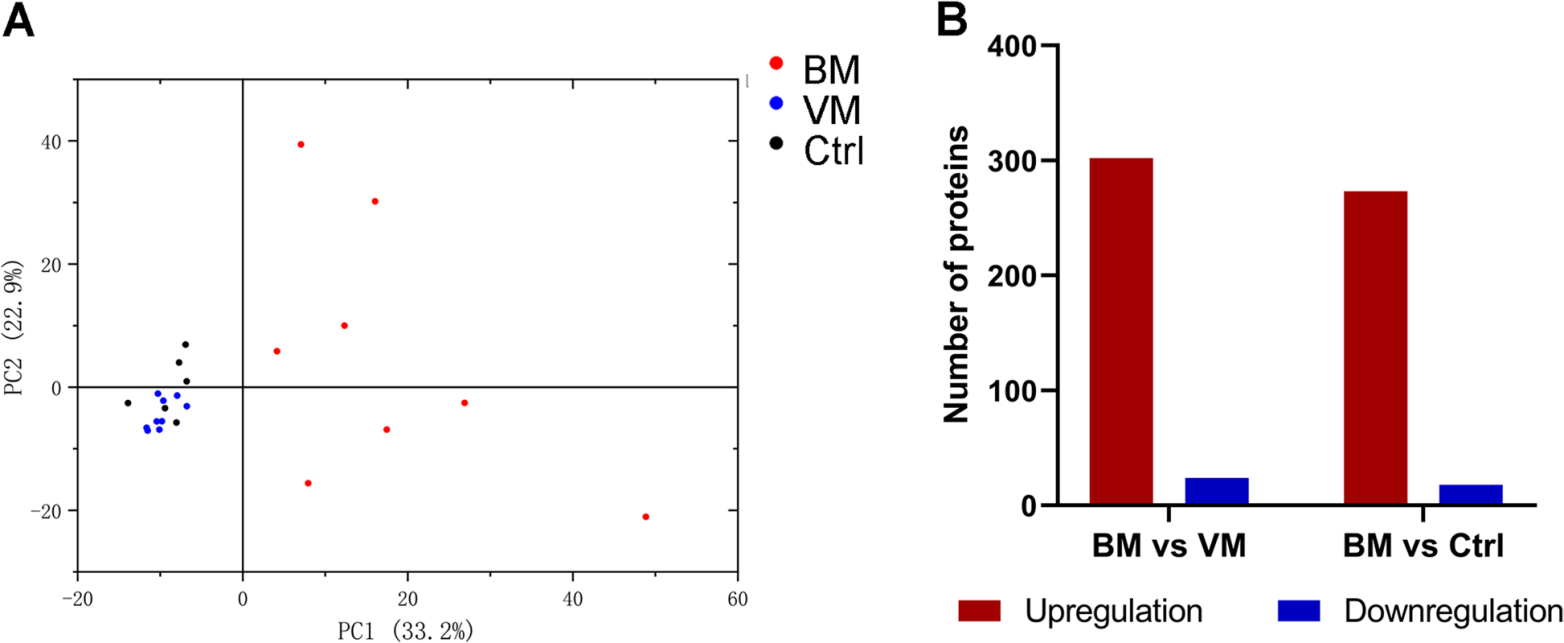
Comparison of CSF proteome profiles between the BM and non-BM groups. **A** Two-dimensional scatter plot of principal component analysis (PCA) distribution of all samples using quantified proteins; **B** Histogram of the number distribution of DEPs in different comparison groups

A total of 10,467 peptides were identified by spectral analysis, of which 8,167 were specific peptides. A total of 1,121 proteins were identified, of which 809 were quantifiable (with quantitative proteins being those proteins for which at least one comparison group has quantitative information). PCA was conducted based on the quantitation of the CSF proteins of each patient, and the PCA graph showed that all patients in the BM group were distinguished from the those of the other two patient groups (Fig. 2A). The quantitative data set is shown in Table S1, additional file 1. Because the protein level of CSF varies extensively, we normalized the relative quantitation of the proteins to the CSF volume. A total of 302 proteins were significantly upregulated and 24 proteins were downregulated in the BM group compared with the VM group, while 273 proteins were significantly upregulated and 18 proteins were downregulated in the BM group compared with the noninfected control group (Fig. 2B).

### Analysis of differentially expressed CSF proteins between the BM and non-BM groups

**Fig. 3:**
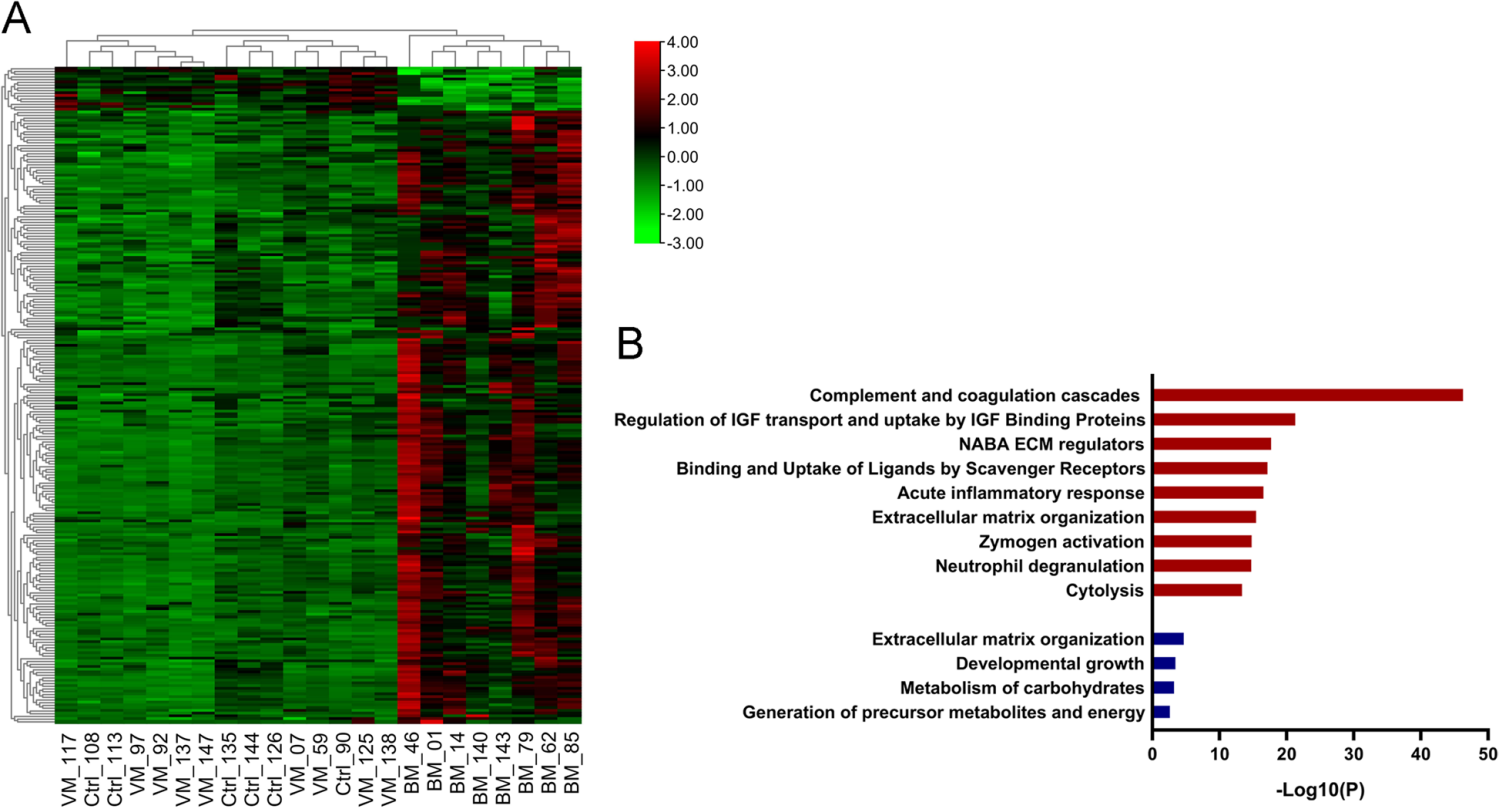
Heatmap of the DEPs between the BM and non-BM groups and bar graph of the top enriched biological functions of these DEPs in both comparison groups

**Fig. 4:**
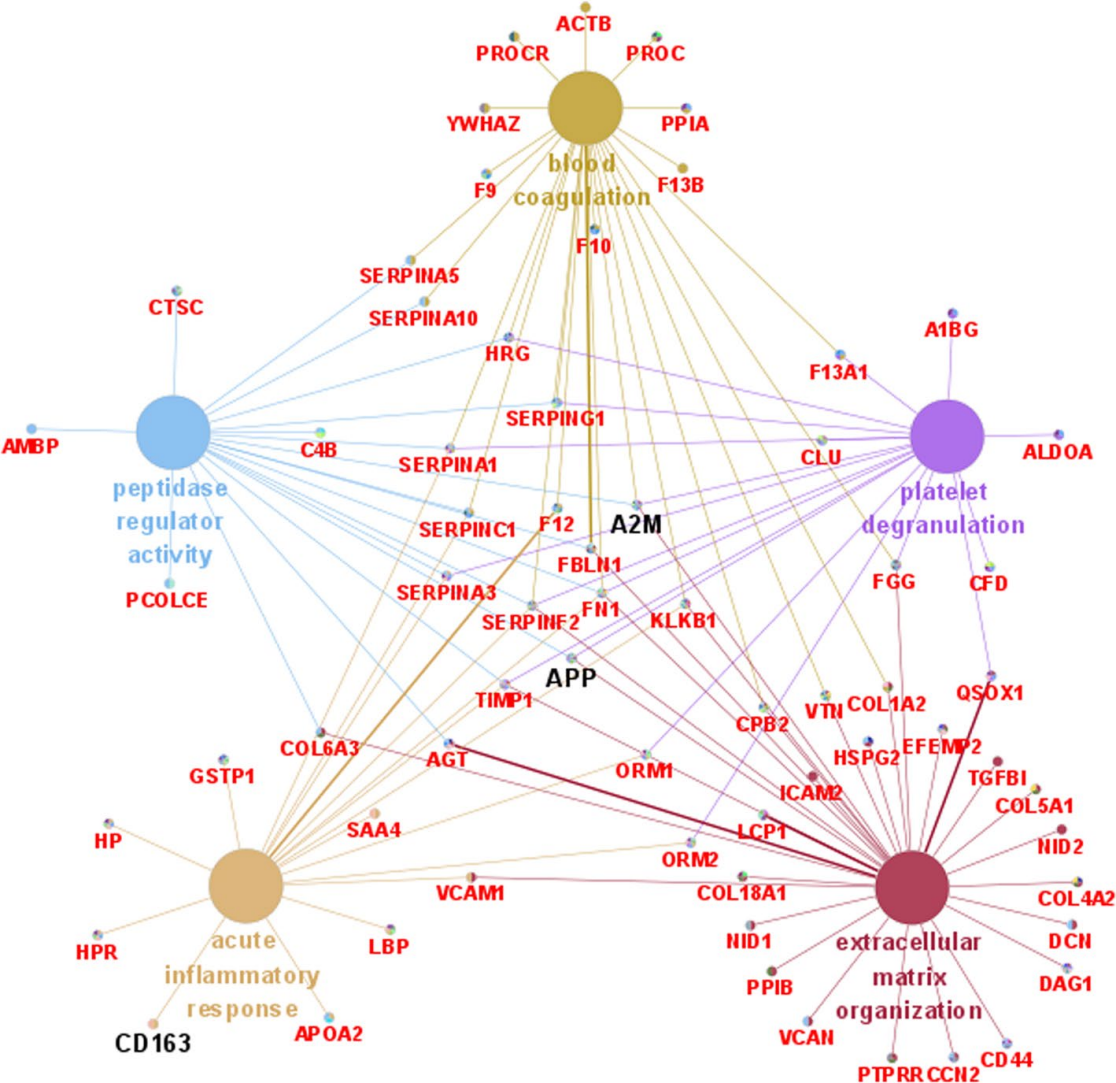
Visualization of the network of major enriched functions and their associated genes

To identify BM-specific proteins, we filtered the DEPs in the BM/VM or BM/control comparisons with stricter criteria. The DEPs in CSF between the BM and non-BM groups were defined according to the following criteria: 1) fold changes in both the BM/VM and BM/control comparisons of more than 2.5 or smaller than 0.67 and *p* < 0.05 and 2) detection in at least 6 BM samples, 6 VM samples and 4 control samples. Based on these criteria, a total of 171 proteins were identified as DEPs between the BM group and non-BM groups: 157 significantly upregulated proteins and 14 significantly downregulated proteins. Based on the expression levels of these 171 proteins, BM patient samples were obviously distinguished from the VM and control groups in the heatmap (Fig. 3A). GO annotation and biological function and pathway enrichment analyses of these 171 proteins were performed on the Metascape online platform. The significantly upregulated proteins in BM were mainly enriched in the functions complement activation, platelet degranulation, regulation of IGF transport and uptake by IGF-binding proteins, acute inflammatory response and extracellular matrix organization, and neutrophil degranulation, while the downregulated proteins in BM were enriched in the terms developmental growth, metabolism of carbohydrates and generation of precursor metabolites and energy (Fig. 3B). The major enriched biological functions with the associated genes were visualized with Cytoscape and are shown in Fig. 4 as a network. In the network, the major biological process terms platelet degranulation, blood coagulation, extracellular matrix organization, acute inflammatory response and peptidase regulator activity are strongly connected by node proteins, such as APP, A2M and SERPINA3. In addition, the node proteins A2M, APP and CD163 were among the top 30 most enriched DEPs between the BM and non-BM groups (Table 3). A2M, which was upregulated in the BM group, is associated with extracellular matrix organization, platelet degranulation, blood coagulation and peptidase regulator activity. The APP protein was downregulated in the BM group and is associated with extracellular matrix organization, platelet degranulation and peptidase regulator activity. CD163 is an acute phase protein and was highly upregulated in the CSF of the BM group.

**Table 3:** Top 30 DEPs between the BM and non-BM groups

Gene name	Protein description	BM/VM	*P* value	BM/Ctrl	*P* value
LBP	Lipopolysaccharide-binding protein	34.7476	0.0006	27.9315	0.0030
HPR	Haptoglobin-related protein	34.1995	0.0005	44.0382	0.0004
CD163	Scavenger receptor cysteine-rich type 1 protein M130	19.7130	0.0002	19.6556	0.0002
FCGBP	IgGFc-binding protein	19.3185	0.0001	20.5446	0.0001
VSIG4	Low affinity immunoglobulin gamma Fc region receptor III-A	17.1259	0.0001	16.2035	0.0002
FCGR3A	Low affinity immunoglobulin gamma Fc region receptor III-A	13.7149	0.0000	15.7777	0.0000
FGG	Fibrinogen gamma chain	13.5045	0.0006	14.1276	0.0008
APOM	Apolipoprotein M	12.4151	0.0021	10.9577	0.0040
HP	Haptoglobin	12.3086	0.0276	13.1471	0.0192
F13A1	Coagulation factor XIII A chain	11.4554	0.0044	10.7878	0.0046
CPN2	Carboxypeptidase N subunit 2	10.9681	0.0004	11.8880	0.0000
SERPINA3	Alpha-1-antichymotrypsin	10.2239	0.0020	11.0671	0.0014
CD14	Monocyte differentiation antigen CD14	10.0526	0.0000	11.2716	0.0000
CD5L	CD5 antigen-like	9.9693	0.0016	13.6411	0.0000
A2M	Alpha-2-macroglobulin	9.9481	0.0000	10.3473	0.0000
CNDP1	Beta-Ala-His dipeptidase	0.1948	0.0012	0.2300	0.0106
FAT2	Protocadherin Fat 2	0.2882	0.0020	0.2801	0.0014
CPE	Carboxypeptidase E	0.2943	0.0003	0.3083	0.0006
KLK6	Kallikrein-6	0.3063	0.0016	0.3328	0.0064
TPI1	Triosephosphate isomerase	0.4314	0.0109	0.4548	0.0244
IGSF8	Immunoglobulin superfamily member 8	0.4340	0.0001	0.4832	0.0222
CPQ	Carboxypeptidase Q	0.4603	0.0003	0.4523	0.0159
TPP1	Tripeptidyl-peptidase 1	0.4745	0.0007	0.4844	0.0056
ADAM22	Disintegrin and metalloproteinase domain-containing protein 22	0.5135	0.0073	0.5476	0.0208
SOD3	Extracellular superoxide dismutase [Cu–Zn]	0.5491	0.0024	0.5373	0.0075
DCN	Decorin	0.5561	0.0060	0.5824	0.0200
APP	Amyloid-beta precursor protein	0.5879	0.0329	0.6077	0.0426
CPVL	Probable serine carboxypeptidase CPVL	0.6167	0.0057	0.6104	0.0033
MDH1	Malate dehydrogenase, cytoplasmic	0.6205	0.0062	0.5937	0.0046
CTSD	Cathepsin D	0.6220	0.0039	0.6290	0.0104

### Verification of potential biomarker candidates for paediatric BM

**Fig. 5:**
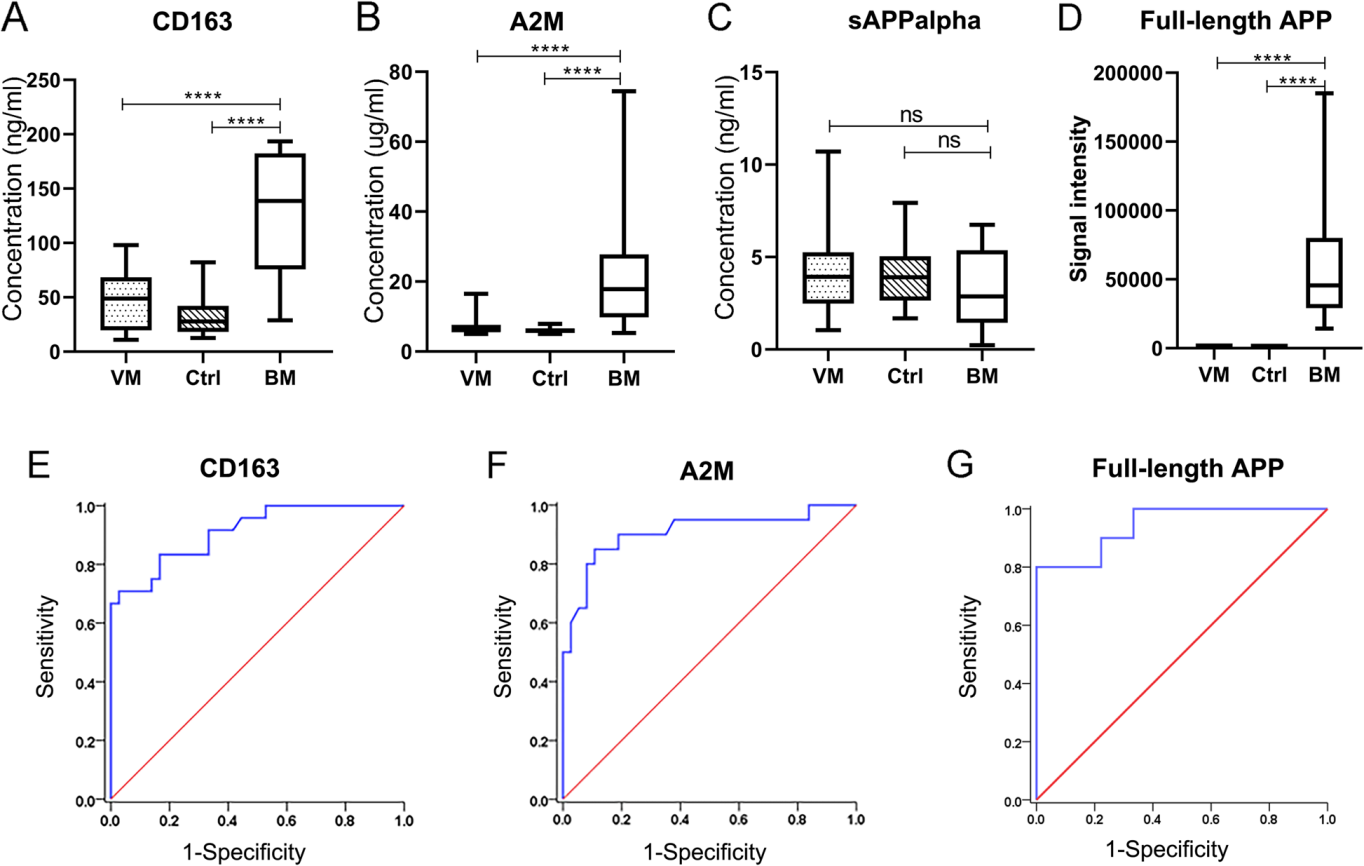
Validation of the protein expression levels of CD163, A2M, sAPPα and full-length APP in CSF and evaluation of their diagnostic performance in paediatric BM

Diagnostic biomarker candidates of A2M, sAPPα and CD163 for paediatric BM were validated by ELISA or western blot in cohorts 1 and 2. The ELISA results showed that the CSF levels of CD163 and A2M in BM patients were significantly higher than those in VM patients and noninfected control patients, while the CSF levels of sAPPα in the BM group were only slightly lower than those in the VM and control groups, and the differences were not significant (Fig. 5 A-D). However, full-length APP was significantly upregulated in the BM group compared with the non-BM groups, as shown by western blot analysis (Additional file 2 – Figure S1).

To evaluate the diagnostic capacity of CSF levels of CD163, A2M and full-length APP in paediatric BM, their specificity and sensitivity as diagnostic biomarkers were determined by ROC curve analysis (Fig. 5 E–G). When the cut-off value of CD163 was set as 87.68 ng/ml, the sensitivity and specificity of CD163 for paediatric BM reached 70.8% and 97.2%, respectively, and the AUC was 0.911 (95% CI: 0.839–0.984). A2M also showed good diagnostic capacity, with an AUC of 0.908 (95% CI: 0.816–1.000). When the cut-off value of A2M was set as 8.05 µg/ml, its specificity and sensitivity were 85% and 89.2%, respectively. Full-length APP could also distinguish BM from non-BM patients, with an AUC value of 0.944 (95% CI: 0.86, 1.000).

## Discussion

### CD163

CD163 is a member of the scavenger receptor cysteine-rich (SRCR) superfamily class B, which is highly expressed on resident tissue macrophages in vivo. Serum CD163 has been demonstrated to act as a macrophage receptor for haemoglobin-haptoglobin complexes and to lead to their phagocytosis, protecting tissues from free haemoglobin-mediated oxidative damage [17–19]. Interestingly, CD163 can directly function as a macrophage receptor for bacteria and can bind both gram-positive and gram-negative bacteria by a cell-binding motif in its second scavenger domain. In addition, the expression of CD163 in monocytic cells has been shown to promote bacteria-induced proinflammatory cytokine production [20]. Therefore, the expression of CD163 is strongly associated with bacterial infection. This association may explain why CD163 in CSF was highly upregulated in BM patients compared with non-BM patients and suggests that CSF CD163 may act as an innate immune sensor and inducer of local inflammation during BM.

Although the diagnostic potential of serum CD163 for bacterial infection has been investigated, CSF CD163 has yet to be evaluated for its diagnostic potential for BM. In one study, the diagnostic value of serum soluble CD163 was evaluated and compared with that of CRT and PCT for meningitis in an observational cohort consisting of 55 patients suspected of having meningitis. Elevated serum levels of sCD163 were the most specific marker for distinguishing bacterial infection from nonbacterial disease, with a high specificity of 0.91 but a low sensitivity of 0.47. However, the overall diagnostic accuracies of CRP (AUC = 0.91) and PCT (AUC = 0.87) were superior (*p* < 0.02 and *p* < 0.06) to that of sCD163 (AUC = 0.72). None of the markers was useful as an independent tool for the clinical diagnosis of purulent meningitis [21]. In our study, we found that elevated sCD163 CSF levels could distinguish bacterial infection from non-BM disease with a high AUC value of 0.911 (95% CI: 0.839–0.984). Moreover, compared with the serum level of sCD163, the CSF level of sCD163 had much better diagnostic sensitivity and specificity, reaching 70.8% and 97.2%, respectively. Therefore, CSF-soluble CD163 is an excellent potential biomarker for paediatric BM. Whether this diagnostic biomarker is also applicable to adult BM needs to be determined.

### A2M

A2M is another top upregulated protein in BM patients compared to non-BM patients. A2M is a homotetrameric glycoprotein, a key member of the alpha macroglobulin superfamily and a major antiproteinase plasma protein. In particular, it is able to inhibit all four kinds of proteases without the direct blockage of the protease active site [22]. In addition, it can regulate the binding of transferrin to its surface receptor; bind defensin and myelin basic protein; and bind several important cytokines, including basic fibroblast growth factor (bFGF), platelet-derived growth factor (PDGF), nerve growth factor (NGF), interleukin-1b (IL-1b), and interleukin-6 (IL-6), and modify their biological activity [22]. The plasma levels of A2M-containing microparticles have been reported to be higher in survivors of sepsis than in either nonsurvivors or healthy volunteers [23, 24]. Compared to the administration of PBS or soluble A2M, administration of A2M-enriched microparticles in mice with microbial sepsis led to a significant reduction in 4-day mortality and significantly reduced the levels of proinflammatory lipid mediators, including PGD2 (~ 20–40%, *P* < 0.05) and the potent chemoattractant LTB4 (> 50%; *P* < 0.05). In vitro, A2M promoted neutrophil–endothelial adhesion; enhanced bacterial phagocytosis, reactive oxygen species production, and cathelicidin release; prevented endotoxin-induced CXCR2 downregulation and preserved neutrophil chemotaxis in the presence of LPS. Therefore, A2M-enriched microparticles in plasma play an important host-protective role in sepsis [24].

Although A2M seems to be associated with the outcome of sepsis, there have been no previous reports on the diagnostic value of A2M in BM, sepsis, or any other infection. In this study, we found for the first time that the CSF level of A2M showed good diagnostic capacity for paediatric BM in a cohort of 62 patients, with an AUC of 0.908 (95% CI: 0.816–1.000). At the cut-off value of 8.05 µg/ml, the specificity and sensitivity of A2M were 85% and 89.2%, respectively.The results suggest that a high CSF level of A2M is more likely to be associated with paediatric BM than with VM or noninfection. For this interesting protein, validation in a multicentre cohort of BM patients, not only in children but also in adults, is strongly recommended.

### APP

APP is a well-characterized causative protein of Alzheimer’s disease. It can be cleaved by α-, β- and γ-secretases, which results in the secretion of sAPPα, soluble APPβ and Aβ_1-42_. According to the quantitative MS data in this study, the CSF level of total APP, including APP and its metabolites, was lower in the BM patients than in the non-BM patients (Table 3). However, the CSF level of full-length APP level was much higher while soluble APPα in CSF was lower in the patients with BM than in the non-BM patients. A possible reason for the reduced metabolism of full-length APP in the patients with BM was a decreased activity of secretases. Visualization of the network of enriched functions revealed that APP is associated with peptidase regulator activity. A2M was highly upregulated in the BM patients (Table 3). A2M can inhibit all four kinds of proteases, and it has been experimentally confirmed that A2M directly interacts with APP [25]. Therefore, we speculate that CSF peptidases were more strongly inhibited in the BM patients than in the non-BM patients. It has been reported that neuroinflammation can cause reduced levels of APP derivatives, such as Aβ, sAPPα and sAPPβ [26, 27]. Krut et al*.* found reduced levels of Aβ, sAPPα and sAPPβ in BM patients compared with AD patients and healthy controls [28]. In this study, we investigated the altered metabolism of CSF APP among patients with BM, patients with VM and noninfected hospital controls. We found that the CSF level of sAPPα in the patients with BM was slightly lower than that in the patients with VM and the noninfected controls, but the differences were not significant. However, western blot showed that the CSF level of full-length APP was much higher in the patients with BM than in those with VM and the noninfected controls. To our knowledge, there are no reports of full-length APP as a diagnostic marker for BM. We recommended validation of full-length APP in CSF in a multicentre cohort.

## Conclusions

Our proteomic analysis provided comprehensive insight into the complex, heterogeneous host response to paediatric BM and allowed the identification of critical differences between BM and non-BM groups. We found that compared with non-BM, paediatric BM was characterized by upregulation of complement and coagulation cascades, regulation of IGF transport and uptake by IGF-binding proteins, the acute inflammatory response and the downregulation of developmental growth and carbohydrate metabolism. Moreover, the CSF levels of CD163, A2M and full-length APP showed excellent diagnostic performance for paediatric BM, with AUC values of 0.911 (95% CI: 0.839–0.984), 0.908 (95% CI: 0.816–1.000) and 0.944 (95% CI: 0.86–1.000), respectively. Among them, A2M and full-length APP are reported for the first time as diagnostic biomarkers of BM, and the findings imply that peptidase regulator activity plays an important role in BM. However, a multicentre cohort study with a large sample size is recommended for validation of these potential diagnostic markers in children and adults with BM.

## Supplementary Information

**Additional file 1: Table S1.**

**Additional file 2: Figure S1**. Detection of full-length APP levels in the patients’ CSF by western blot.

## Data Availability

The LC–MS/MS proteomics data have been deposited in the ProteomeXchange Consortium via the PRIDE [29] partner repository with the dataset identifier PXD026663.
