# Supplementary material for: Quantitative proteomic analysis of cerebrospinal fluid reveals CD163, A2M and full-length APP as potential diagnostic biomarkers of paediatric bacterial meningitis
Source: Proteome Sci. 2022 May 6;20:8. doi: 10.1186/s12953-022-00191-5 (PMC9074227; doi:10.1186/s12953-022-00191-5)
Supplement: Supplementary file 2 — Additional file 2: Figure S1. Detection of full-length APP levels in the patients’ CSF by western blot. [file 12953_2022_191_MOESM2_ESM.docx]

**Supplement-Figure**

**Figure S1**


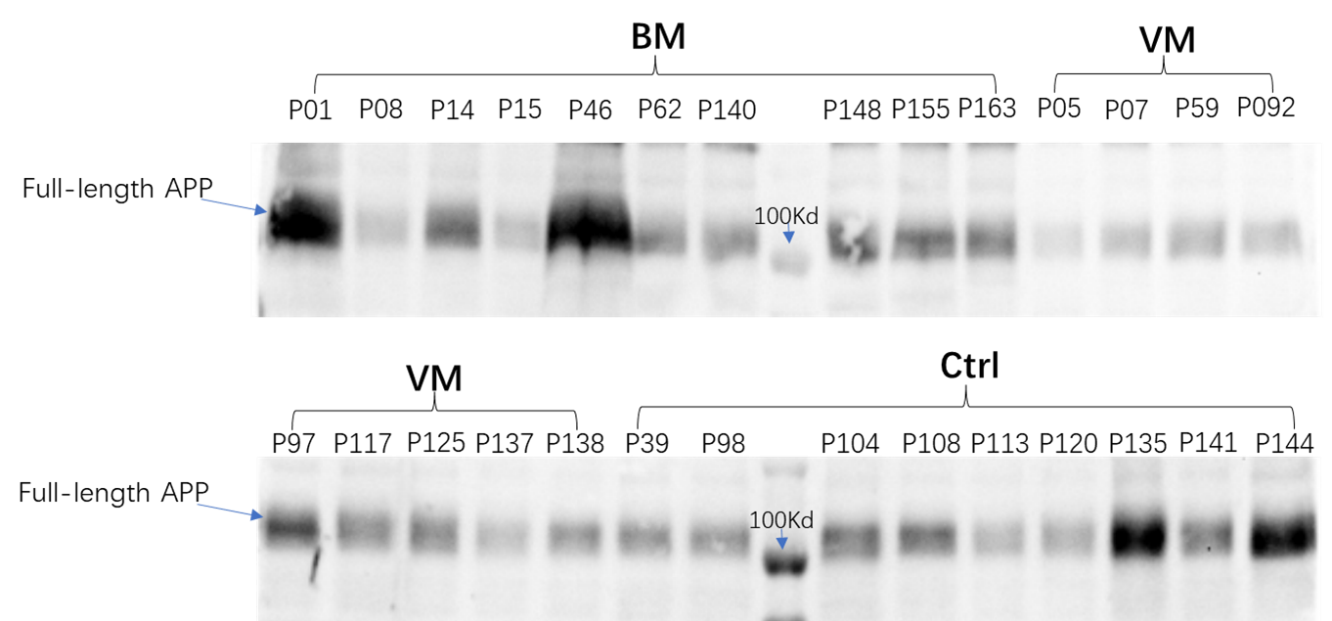


**Figure S1: Detection of full-length APP levels in the patients’ CSF by western blot**
